# Lifestyle Intervention Therapy Modulates Global DNA Methylation and Adipogenic Gene Expression in Severely Obese Hypogonadal Men

**DOI:** 10.3390/metabo16030198

**Published:** 2026-03-16

**Authors:** Siresha Bathina, Virginia Fuenmayor Lopez, Mia Prado, Salina Biene Teo, Dennis T. Villareal, Rui Chen, Clifford Qualls, Reina Armamento-Villareal

**Affiliations:** 1 Division of Endocrinology Diabetes and Metabolism, Baylor College of Medicine, Houston, TX 77030, USA; 2 Department of Medicine, Michael E. DeBakey Veterans Affairs (VA) Medical Center, Houston, TX 77030, USA; 3 Office of Research, New Mexico VA Health Care System, Albuquerque, NM 87108, USA

**Keywords:** obesity, hypogonadism, lifestyle intervention, DNA methylation

## Abstract

**Background/Objectives:** Previous studies have suggested that lifestyle intervention (LSI) therapies involving diet and exercise can modulate DNA methylation; however, whether this occurs in severely obese hypogonadal men undergoing weight loss from diet and exercise remains unclear. **Methods:** In this study, we investigated the effects of weight loss from diet and exercise on global DNA methylation as well as on the mRNA expression of specific demethylation enzymes, *DNMT1*, *DNMT3A*, and *DNMT3B*—in peripheral blood mononuclear cells (PBMCs) and DNA methylation markers in DNA of severely obese hypogonadal men. This is a secondary analysis of samples of severely obese (body mass index of ≥35 kg/m^2^) hypogonadal men undergoing weight loss from diet and exercise in addition to an aromatase inhibitor (anastrozole) or placebo for a total of 12 months. **Results:** LSI therapy significantly reduced global DNA methylation and 5-methylcytosine (5-mC) levels, decreased *DNMT1*, *DNMT3A*, and *DNMT3B* (*p* < 0.05) mRNA levels and markedly decreased *CEBPα*, *FTO*, and *PPARγ* mRNA expression. The reduction in global methylation was independent of aromatase inhibitor use. **Conclusions:** In summary, our findings suggest that LSI induces epigenetic modifications in leukocytes, possibly through the regulation of *DNMT* gene expression. Future studies are warranted to clarify the mechanistic pathways linking lifestyle-induced epigenetic alterations to metabolic health outcomes.

## 1. Introduction

Obesity has become a global epidemic, contributing to a wide spectrum of metabolic and endocrine disorders including type 2 diabetes mellitus, cardiovascular disease, and male hypogonadism [1,2,3]. The recent literature suggests that obesity and related metabolic dysfunctions are accompanied by alterations in epigenetic regulation, particularly DNA methylation [4], which may mediate the interaction between environmental and genetic factors [5,6].

DNA methylation, the covalent addition of a methyl group to cytosine residues within CpG dinucleotides, is one of the most studied epigenetic modifications regulating gene expression [7]. This process is catalyzed by DNA methyltransferases (*DNMTs*), including *DNMT1*, *DNMT3A*, and *DNMT3B*, which play distinct roles in maintaining and establishing methylation patterns [8]. Aberrant DNA methylation has been implicated in several obesity-related pathologies, influencing genes involved in lipid metabolism, adipogenesis, and insulin sensitivity [9]. Specifically, the peroxisome proliferator-activated receptor gamma (*PPARγ*) and CCAAT/enhancer-binding protein alpha (*CEBPα*) are critical transcription factors governing adipocyte differentiation and glucose homeostasis, and their epigenetic regulation has been associated with obesity and metabolic dysfunction [10,11]. Our group showed that combined diet and exercise resulted in greater improvement in physical function [12] and improvement in metabolic profile [13] than diet or exercise alone. Beyond improving body composition and metabolic health, Keller et al.’s studies on 120 adults (BMI: ~30 kg/m^2^) in an 18-month diet +/− physical activity lifestyle randomized trial found that successful weight loss was associated with specific genome-wide DNA methylation changes in blood [14]. Aerobic exercise has been reported to modify DNA methylation in skeletal muscle, adipose tissue, and leukocytes, leading to altered expression of metabolic and inflammatory genes [15]. Dietary factors such as caloric restriction and nutrient composition can also influence methylation status by modulating one-carbon metabolism and methyl donor availability [16]. Despite these insights, the combined effects of diet and exercise on DNA methylation in severely obese hypogonadal men remain largely unexplored.

A prior study in obese individuals found that increased global DNA methylation and elevated *DNMT1* expression have been associated with systemic inflammation and insulin resistance [17]. Conversely, in a rat study, lifestyle modification has been linked to reduced *Dnmt* activity and demethylation of genes regulating metabolism and inflammation [18]. However, most of these findings are derived from pre-clinical and general obese patients, but specific epigenetic mechanisms through which lifestyle interventions (LSIs) influence metabolic outcomes in severely hypogonadal men have not been clearly elucidated. Given these gaps in knowledge, the present study aimed to investigate the effects of LSIs by diet and exercise to promote weight loss on global DNA methylation in peripheral blood mononuclear cells (PBMCs) of obese hypogonadal men. We focused on the methylation and expression of *PPARγ* and *CEBPα* as representative metabolic regulators, and the expression of *DNMT1*, *DNMT3A*, and *DNMT3B* as key enzymes of DNA methylation machinery. By integrating molecular and physiological data, this study seeks to provide mechanistic insights into how LSIs modulate epigenetic pathways which may ultimately contribute to improved metabolic and endocrine function in obese hypogonadal men.

## 2. Materials and Methods

### 2.1. Study Design and Patient Population

This was a secondary analysis of DNA and RNA samples obtained from participants of the study, titled “Aromatase inhibitors and weight loss in severely obese men with hypogonadism” (NCT03490513). This study was a randomized double-blind placebo-controlled trial on the effect of weight loss from LSI in combination with an aromatase inhibitor (LSI + AI), anastrozole 1 mg daily, versus placebo (LSI + PBO) over 1 year on hormonal profile and symptoms of hypogonadism in men with severe obesity and hypogonadism. Inclusion/exclusion criteria were as previously published [19], but briefly, the study recruited severely obese men (BMI of ≥35 kg/m^2^), 35–65 years old, with an average fasting total T done twice between 8 a.m. and 10 a.m. on 2 separate days within 1 month of <300 ng/dL, with luteinizing hormone (LH) of <9.0 mIU/L and estradiol (E2) of ≥14 pg/mL, and with symptoms consistent with hypogonadism. The exclusion criteria included the following: (1) clinical/biochemical evidence of hypothalamic/pituitary disease; (2) drugs affecting gonadal hormone levels, production and action, or bone metabolism (bisphosphonates, teriparatide, denosumab, glucocorticoids, and phenytoin); (3) diseases affecting bone metabolism (e.g., hyperparathyroidism, untreated hyperthyroidism, osteomalacia, chronic liver disease, significant renal failure, hypercortisolism, malabsorption, immobilization, and Paget’s dis.); (4) prostate carcinoma or elevated serum PSA > 4 ng/mL; (5) hematocrit (HCT) more than 50%; (6) untreated severe obstructive sleep apnea; (7) cardiopulmonary disease (e.g., myocardial infarction within 6 months, unstable angina, and stroke) or unstable disease (e.g., NYHA Class III or IV congestive heart failure, severe pulmonary disease requiring steroid pills or the use of supplemental oxygen that would contraindicate exercise or dietary restriction); (8) unstable weight (i.e., ±2 kg) in the last 3 months; (9) BMD T-score of less than −2.0 at the spine, femoral neck, or total femur; (10) T2DM with fasting blood glucose of >160 mg/dL or A1C of >9.5%.

This study was conducted at Michael E. DeBakey VA Medical Center and the protocol was approved by the Institutional Review Board of Baylor College of Medicine. All participants provided written informed consent in accordance with the guidelines in the Declaration of Helsinki for the ethical treatment of human subjects. This study started in May 2018 and ended in June 2025.

**Figure 1:**
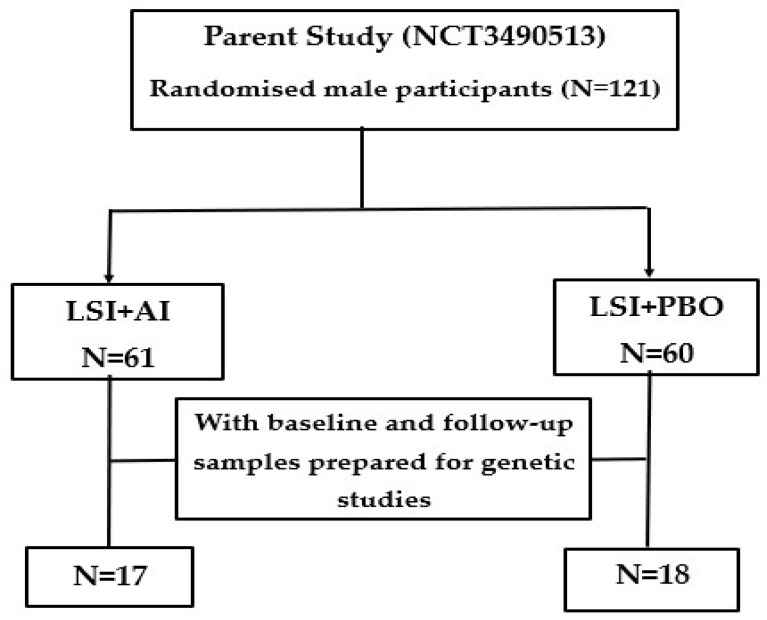
Flow diagram of participants included in the study. LSI + AI—lifestyle intervention + aromatase inhibitor; LSI + PBO—lifestyle intervention+ placebo.

This specific study focused on the methylation changes over time on a subset of subjects with DNA and RNA taken at least at 2 timepoints during the intervention (please see Figure 1 below). 

### 2.2. Diet and Exercise Intervention

Dietary intervention was managed with the aid of a dietitian. Participants were instructed to consume a balanced diet to provide a deficit of 500–750 kcal/day from daily energy requirement. Follow-up visits with dietitian were weekly for the first 3 months and then every 2 weeks thereafter.

Exercise training was supervised by an exercise physiologist and consisted of aerobic training for ~45 min in duration and involved walking on a treadmill and stationary cycling. The resistance training involved nine upper-extremity and lower-extremity exercises with the use of weight-lifting machines. Initially, participants exercised in-person at our facility twice a week. The participants were also instructed to perform home-based exercises involving ground walking, treadmill walking, or stationary biking if available as well as resistance exercise using body weight (e.g., abdominal crunches), resistance bands, and ankle weights. However, because of COVID-19 pandemic restrictions, patients were later allowed to perform exercises in a gym of their choice paid for the study or just do home exercises using as much as possible the same routine.

### 2.3. Body Mass Index (BMI)

Body weight and height were measured by a standard weighing scale and stadiometer, respectively. BMI (kg/m^2^) was calculated by dividing the weight (in kilograms) by height (in meters) squared.

### 2.4. Gene Expression Studies

Blood samples were collected early in the morning after an overnight fast and processed, and then, the samples were stored at −80 °C until analysis. PBMCs were isolated from whole blood using Ficoll density gradient centrifugation. The PBMC fraction contained lymphocytes (T cells, B cells, and NK cells) and monocytes but excluded granulocytes such as neutrophils. Gene expression of *PPARγ*, *CEBPa*, *FTO*, *DNMT1*, *DNMT3A*, and *DNMT3B* in PBMCs was performed by real-time quantitative polymerase chain reaction at baseline (BL) and at 12 months (12 M).

#### 2.4.1. RNA Extraction and qPCR Studies

RNA was extracted from PBMCs using RiboPure Blood (Invitrogen, Carlsbad, CA, USA #AM1928). A total of 200 ng of RNA was used for retro transcription into cDNA and performed using Superscript VILO Master Mix (Invitrogen, Carlsbad, CA, USA) in triplicates following protocol instructions. FAM-labeled TaqMan gene expression assays (Applied Biosystem, College Station, TX, USA) were used for *PPARγ* (Assay ID:), *CEBPα* (Hs00269972_s1:), *FTO* (Hs01057145_m1), *DNMT1* (Hs00945875_m1), *DNMT3A* (Hs01027162_m1), and *DNMT3B* (Hs00171876_m1), and a VIC-labeled TaqMan gene expression assay for housekeeping *18S* (assay ID: Hs03928990_g1). TaqMan Universal Master Mix was used following the manufacturer’s protocol. Please see the supplementary details of kits and chemicals used in this study.

Relative quantification: Relative quantification of gene expression of our samples was compared with that of human control total RNA, analyzed by TaqMan-based real-time PCR analysis (Applied Biosystems, #4307281, Carlsbad, CA, USA) was calculated using the DDCT method and adjusted for housekeeping gene expression. Data analysis was performed using a real-time PCR system QuantStudio5 and Quant Studio Design & Analysis Software 1.3.1.

#### 2.4.2. DNA Extraction and Methylation Studies

Genomic DNA was isolated from peripheral leukocytes [20] according to the manufacturer’s instructions. DNA quality and quantity were assessed by spectrophotometry (NanoDrop) Bioanalyzer 2100 (Agilent Technologies, Santa Clara, CA, USA). and samples were normalized to 200 ng/µL for methylation analysis. Global DNA methylation (5-methylcytosine, 5-mC) was quantified using the Global DNA Methylation Assay Kit (5-Methyl Cytosine, Colorimetric; Abcam, Cambridge, UK; Cat. No. ab233486) following the manufacturer’s instructions.

Global DNA methylation was expressed as the percentage of 5-methylcytosine (5-mC%) relative to total cytosine content and calculated according to the manufacturer’s instructions. Values are expressed as the fraction of total methylated cytosine. Because the primary objective of this secondary analysis was to evaluate sustained epigenetic changes following long-term lifestyle intervention, we analyzed specimens collected at baseline (BL), 6 months, and 12 months (12 M), representing the full duration of the intervention for global methylation studies.

#### 2.4.3. Statistical Analysis

Overall longitudinal analysis of 5 mC% or global DNA methylation (GM) were performed by repeated measures ANOVA with visit (baseline, 6 months, and 12 months) as the repeated factor adjusted for the baseline outcome value and treatment group (lifestyle with AI or placebo); change in testosterone, estradiol, or weight; or metformin use as covariates. Comparisons of 6- and 12-month values with baseline values for the different methylation parameters were performed by Student’s *t*-test. Data were presented as the means ± SD in tables and text, and the means ± SE in the figures. Data in graphs were analyzed using Prism 9.0 (GraphPad, San Diego, CA, USA) and tables were managed using Excel 2013 (Microsoft, Redmond, WA, USA), analyzed by Statgraphics Centurion XVI X64 (Statgraphics Technologies, Inc., The Plains, VA, USA) and confirmed using SAS version 9.3 (SAS Institute, Inc., Cary, NC, USA) A *p*-value of < 0.05 was considered statistically significant.

## 3. Results

Thirty-five participants with RNA and DNA samples taken at least at 2 timepoints were included in this study (Figure 1). The average baseline weight of these subjects was 135.9 ± 20.5 kg, and the baseline BMI was 43.8 ± 5.3 kg/m^2^. Table 1 below shows the baseline characteristics of the participants included in the study. There were no significant differences in clinical characteristics between those randomized to LSI + AI compared to the LSI + PBO at baseline. In addition, there were no significant differences in baseline characteristics of the entire population and the subjects included in this study (see Supplementary Table S2).

### 3.1. Body Composition

At the end of the study, the subjects lost an average of −3.7 ±3.6% (−6.7 ± 7.1 kg). Testosterone significantly increased in the LSI + AI group compared to the LSI + PBO group (+174.6+/−134.3 ng/dL vs. +43.8+/−103.5 ng/dL; respectively, *p* = 0.01), and estradiol significantly decreased in the LSI + AI group compared to LSI + PBO group (−13.9+/−19.0 pg/mL vs. +1.5+/−7.7 pg/mL; respectively, *p* = 0.01) at the end of 12 months. Table 2 below shows the changes in body composition of the participants compared to baseline at 6 and 12 months. Body fat, visceral adipose tissue, and lean mass (total and appendicular) decreased from baseline. However, these changes were not statistically significant.

### 3.2. DNMTs Expression Is Downregulated Following Diet and Exercise Intervention

**Figure 2:**
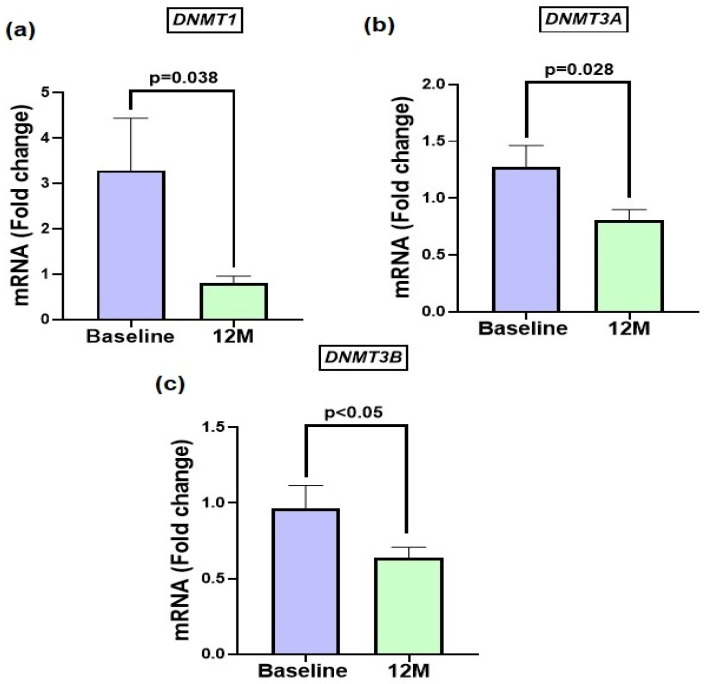
Lifestyle ± aromatase inhibitors downregulate DNA methyltransferase (*DNMT*) gene expression in PBMCs. mRNA expression significantly decreased after 12 months of lifestyle intervention compared with baseline for *DNMT1* (*p* = 0.038) (**a**), *DNMT3A* (*p* = 0.028) (**b**), and *DNMT3B* (*p* < 0.05) (**c**). Data are presented as mean fold change ± SEM.

Lifestyle with or without aromatase inhibitors (LSI ± AI) significantly modulated the expression of DNA methylation-related genes (Figure 2). Reduction in mRNA expression was observed at 12 months compared to baseline for *DNMT1* (BL: 3.29 ± 1.1 vs. 12 M: 0.78 ± 0.16, *p* = 0.038), *DNMT3A* (BL: 1.28 ± 0.18 vs. 12 M: 0.81 ± 0.09, *p* = 0.028), and *DNMT3B* (BL: 0.97 ± 0.15 vs. 12 M: 0.64 ± 0.07, *p* = 0.05) following the intervention (Figure 2a–c). Collectively, these findings demonstrate a consistent downregulation of key components of DNA methylation machinery after the 12-month lifestyle program.

### 3.3. Lifestyle Modification Reduces Global and 5-mC% DNA Methylation

**Figure 3:**
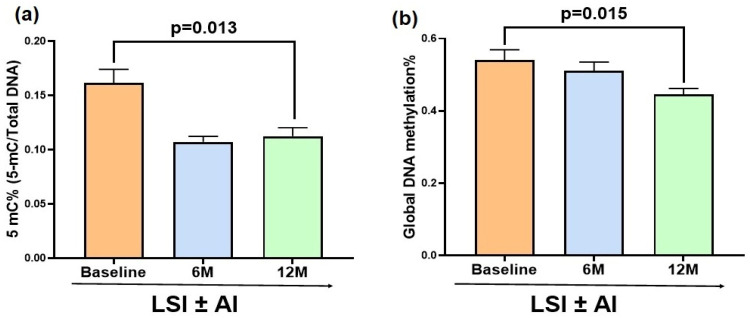
Lifestyle intervention with or without an aromatase inhibitor (LSI ± AI) reduces global DNA methylation and 5-methylcytosine % (5-mC%) levels in DNA of buffy coat samples. There was a decrease from baseline in 5-mC% levels at both follow-up timepoints but significant only at 12 months (*p* = 0.013) (**a**) and global DNA methylation percentage in PBMCs across the same timepoints which was significant only at 12 M (*p* = 0.015) with intervention (**b**). Bars represent the mean ± SEM.

To validate whether the transcriptional changes were accompanied by epigenetic alterations at the DNA level, global DNA methylation markers were assessed in the PBMC samples collected at baseline (BL), 6 months (6 M), and 12 months (12 M). LSI ± AI significantly reduced the percentage of 5-mC%, with levels decreasing from BL (0.18 ± 0.03) to 6 M (0.103 ± 0.005; *p* = 0.052) and 12 M (0.081 ± 0.004; *p* = 0.013) (Figure 3a). Similarly, global DNA methylation showed a downward trajectory, declining from BL (0.85 ± 0.18) to 6 M (0.49 ± 0.03; *p* = 0.053) and 12 M (0.39 ± 0.014; *p* = 0.015) (Figure 3b). Collectively, these data indicate that LSI ± AI promotes gradual genomic DNA demethylation in PBMCs over the 12-month intervention period.

To examine the influence of AI, as half of the subjects on this study were on it and hormone studies showed significant differences in testosterone and estradiol levels between those who were and were not on AI, we compared the change in 5-mC% and global methylation at 6 and 12 months between the two groups. We found significant differences over time (6 months and 12 months; *p* = 0.002 for 5-mC% and *p* < 0.001 for global methylation by repeated measures ANOVA) but no differences due to treatment with AI or PBO (*p* = 0.12 for 5-mC% and *p* = 0.06 for global methylation), testosterone (*p* = 0.62 for 5-mC% and *p* = 0.21 for global methylation), or estradiol (*p* = 0.35 for 5-mC% and *p* = 0.92 for global methylation); and an independent difference due to weight (*p* = 0.02 for 5-mC% and *p* = 0.03 for global methylation). Since some of our patients were on metformin which has been known to affect methylation, we adjusted for the intake of the drug which did not show any difference between those on LSI + AI and LSI + PBO (Table 3).

### 3.4. Lifestyle Intervention Alters Expression of Adipogenic and Metabolic Genes

**Figure 4:**
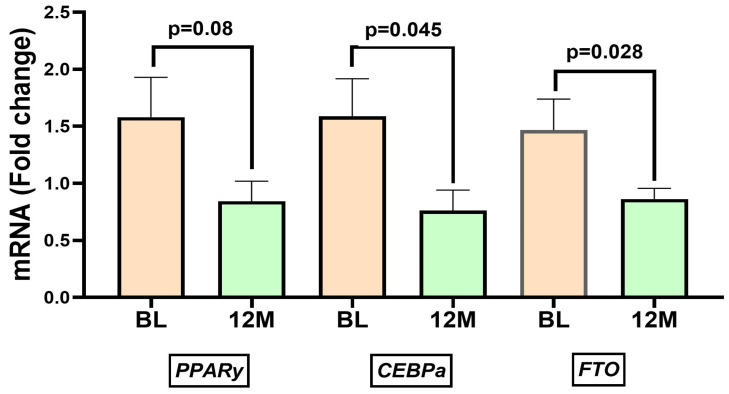
Lifestyle intervention with or with aromatase inhibitors (LSI ± AI) alters expression of adipogenic and metabolic genes. mRNA expression levels of *PPARγ*, *CEBPα*, and *FTO* were measured in PBMCs before (BL/baseline) and after the lifestyle intervention (12 M). Bars represent the mean fold change ± SEM. Expression of all three genes significantly decreased following the intervention, indicating reduced adipogenic and metabolic activity associated with LSI ± AI.

LSI ± AI significantly modulated the expression of genes associated with adipogenesis and metabolism in PBMCs in men with severe obesity and hypogonadism. LSI ± AI also decreased mRNA levels of adipogenic markers (Figure 4), *PPARγ* (BL: 1.53 ± 0.37 vs. 12 M: 0.83 ± 0.18; *p* = 0.08), and *CEBPα* (BL: 1.59 ± 0.32 vs. 12 M: 0.76 ± 0.18; *p* = 0.045). Similarly, *FTO* mRNA expression was significantly decreased after 12 months of LSI ± AI (BL: 1.47 ± 0.27 vs. 12 M: 0.86 ± 0.09; *p* = 0.028) (Figure 4). Notably, the direction of change was consistent across participants, and the coordinated downregulation of *PPARγ*, *CEBPα*, and *FTO* suggests a broad transcriptional response to the LSI ± AI.

## 4. Discussion

This study provides novel evidence that combined diet and exercise elicit significant epigenetic and transcriptional adaptations in severely obese hypogonadal men. We observed reductions in global DNA methylation, decreased expression of DNA methyltransferases (*DNMT1*, *DNMT3A*, and *DNMT3B*), and downregulation of key metabolic genes (*PPARγ*, *CEBPα*, and *FTO*) following 12 months of LSI ± AI. Our analysis also indicated that treatment with AI or PBO showed no influence on the changes in methylation. Thus, our findings suggest that the combination of diet and exercise reprograms the leukocyte methylome, which likely accompanies the metabolic improvement associated with LSI.

DNA methylation, catalyzed by DNMTs, regulates gene expression, genomic stability, and imprinting [7]. Hyperactivation of DNMTs has been linked to obesity, insulin resistance, and chronic inflammation [17]. The human genome encodes five *DNMTs*—*DNMT1*, *DNMT2*, *DNMT3A*, *DNMT3B*, and *DNMT3L*. Among these, *DNMT1*, *DNMT3A*, and *DNMT3B* are canonical cytosine-5 methyltransferases which contain an N-terminal regulatory region and a C-terminal catalytic domain that uses S-adenosylmethionine (SAM) as a methyl donor and a base-flipping mechanism to generate 5-methylcytosine [21], representing global methylation. *DNMT1* preferentially methylates hemi-methylated DNA and is therefore considered the principal maintenance methyltransferase [22], whereas *DNMT3A* and *DNMT3B* primarily act on unmethylated DNA to establish de novo methylation; dysregulation of these enzymes affects adipose biology [23]. 

A prior study by Yang et al. suggested adipocyte differentiation involves large epigenomic changes (histone modifications, DNA methylation) in precursor cells (e.g., 3T3-L1) entering adipogenesis in lean murine adipocytes [24]. While other studies showed elevated *DNMT3A* expression in adipose tissue of transgenic mice [23]; these findings underscore the close association between DNMT activity and adipose tissue biology. Although not significantly different from baseline, our subjects experienced a reduction in body fat (total and visceral) with weight loss. The observed reduction in *DNMT1*, *DNMT3A*, and *DNMT3B* expression (Figure 2) after weight loss from diet and exercise suggests that LSI can downregulate methylation machinery, promoting a more transcriptionally permissive chromatin state. These findings are consistent with earlier studies showing exercise-induced hypomethylation [25] and decreased DNMT expression which may also involve other tissues and cells such as skeletal muscle and leukocytes [15].

Although human intervention studies have not yet directly demonstrated suppression of *DNMT1*, *DNMT3A*, or *DNMT3B* in metabolic tissues following lifestyle or exercise interventions, mechanistic evidence from animal and cellular models shows that reduced *DNMT1* can drive passive demethylation [26], while decreased DNMT3A/3B limits de novo methylation [27]. Exercise-regulated DNMT dynamics in muscle and metabolic tissues have been observed in preclinical models [28], supporting our findings. Such changes can activate transcription of genes involved in mitochondrial biogenesis, oxidative metabolism, and anti-inflammatory responses—pathways central to the metabolic improvements associated with sustained physical activity [29].

Despite the emergence of effective weight loss drugs, exercise and dietary modifications remain important components in the management of obesity [30]. LSI not only improves body composition and glucose homeostasis but also remodels the epigenome [31]. Our data indicate that both global 5-mC and total DNA methylation levels were significantly reduced after LSI over 12 months, which was demonstrated only in the PBMCs in our study as we have no other tissues available. These changes occurred independent of AI use. Our observations agree with the results from the studies of Ronn et al, who analyzed subcutaneous adipose tissue biopsies from 23 previously sedentary, healthy men before and after 6 months of endurance exercise training. In this study, the authors observed widespread genome-wide methylation changes, with 17,975 CpG sites (across 7663 genes) significantly altered after training. Moreover, several loci with methylation changes also showed corresponding mRNA expression shifts, including key obesity and type 2 diabetes-related genes TCF7L2 and KCNQ1, demonstrating coordinated epigenetic and transcriptional remodeling in metabolic tissues [5]. Nitert et al. also showed the impact of an exercise intervention on genome-wide DNA methylation in human skeletal muscle of patients with diabetes [32].

**Figure 5:**
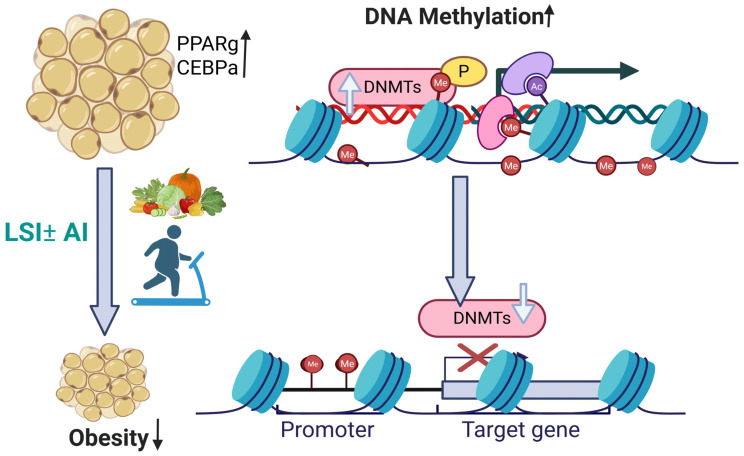
Conceptual mechanism of LSI-induced epigenetic remodeling. The above figure illustrates the proposed mechanism by which lifestyle intervention (LSI) with or without aromatase inhibitors (±AIs) modulates DNA methylation and adipogenic gene regulation. Increased DNA methylation mediated by DNA methyltransferases (*DNMTs*) is shown at regulatory genomic regions, accompanied by histone modifications such as methylation (Me) and acetylation (Ac), leading to altered chromatin structure and transcriptional activity. LSI reduces *DNMT* activity; AI has no independent effect on methylation in this study. This epigenetic remodeling is associated with reduced adipogenesis, reflected by decreased adipocyte accumulation and downregulation of key adipogenic transcription factors, ultimately contributing to reduced obesity.

Based on our findings, we hypothesize that the observed suppression of *DNMT1*, the primary maintenance methyltransferase, may promote passive loss of DNA methylation during cell turnover and concurrent reductions in *DNMT3A* and *DNMT3B* activity, enzymes responsible for de novo methylation, thus further limiting methylation as illustrated in Figure 5. Results from the genome-wide methylation studies of Benton et al. in human adipose tissue also showed differential methylation of obesity-associated genes including *DNMT3A* in obese women before and after gastric bypass [33].

Weight-loss interventions and reductions in inflammation were often accompanied by partial “reversal” of methylation changes, suggesting a link between inflammation driven by adiposity and epigenetic regulation [34]. In our study, we observed transcriptional downregulation of *PPARγ*, *CEBPα*, and *FTO*, highlighting the metabolic reprogramming induced by LSI. *PPARγ* and *CEBPα* are key adipogenic transcription factors that promote lipid accumulation and adipocyte differentiation [35] This suppression following 12 months of LSI indicates reduced adipogenic signaling which may enhance metabolic efficiency. Similar findings have been reported in exercise interventions, where *PPARγ* promoter demethylation correlated with metabolic adaptations in skeletal muscle [25]. The decline in FTO mRNA suggests normalization of the obesity-linked RNA demethylase pathway. FTO regulates energy expenditure and appetite through m6A RNA demethylation; its overexpression is associated with obesity and insulin resistance [36]. Downregulation of FTO following lifestyle modification may contribute to improved metabolic control and energy balance.

## 5. Limitations

This study is limited to using PBMCs as a surrogate for systemic epigenetic profiling. Although peripheral methylation patterns often reflect whole-body metabolic adaptations, tissue-specific analysis (e.g., skeletal muscle or adipose tissue) would provide stronger mechanistic evidence. However, these tissues are not easily obtainable in humans. This is the reason why some epigenetic studies [37,38] have relied on easily accessible surrogate tissues such as PBMCs. Accordingly, cellular heterogeneity within PBMC samples represents a limitation, and the observed methylation changes should be interpreted with caution and considered primarily as signals warranting further investigation using approaches that control for immune cell composition (e.g., cell sorting), rather than definitive conclusion. Nevertheless, our data provide supporting evidence for reduced methylation activity, as we observed decreased expression of *DNMT*s. In addition, this study included only severely obese hypogonadal men; therefore, our findings may not be generalizable to women, eugonadal or less obese populations. Moreover, our cohort size was small and not powered for subgroup analysis; thus, future studies should include larger populations to confirm differential responses. In addition, a locus-specific methylation analysis would have strengthened our data but was not performed in our study. Finally, half of our subjects were on aromatase inhibitors. However, there was no difference in methylation between those on aromatase inhibitors and those on placebo, suggesting that the changes in methylation patterns in the epigenome of our subjects are primarily due to LSI with contribution from the accompanying weight change.

## 6. Conclusions

In summary, weight loss from combined lifestyle therapy induces favorable epigenetic reprogramming in obese hypogonadal men, characterized by reduced global DNA methylation, downregulation of *DNMT*s, and suppression of adipogenic genes. Given the small number of subjects in our study, a study with a larger sample size, a longitudinal analysis beyond 12 months, and assessment of locus-specific methylation to assess the stability of these methylation changes and their relationship to long-term clinical outcomes is necessary to confirm our findings. Furthermore, future work integrating genome-wide methylation, transcriptomic, and metabolomic analyses could unravel specific pathways linking methylation to sustainable metabolic improvement.

## Figures and Tables

**Table 1:** Baseline characteristics of the subjects.

Body Parameter	Anastrozole (n = 17)	Placebo (n = 18)	*p*-Value
**Age**	54.1 ± 5.2	49.8 ± 8.0	0.07
**BMI**	42.3 ± 5.9	44.4 ± 4.5	0.26
**Weight**	131.0 ± 22.4	140.8 ± 16.0	0.15
**Racial background**			0.30
**White**	7	10	
**Blacks**	10	7	
**Type 2 diabetes**	11	7	0.17
**Metformin**	7	7	0.85
**Hemoglobin A1c**	6.9 ± 1.1	6.8 ± 1.4	0.92
**Testosterone**	221.5 ± 47.8	235.1 ± 47.5	0.41
**Estradiol**	26.3 ± 17.9	21.4 ± 5.0	0.29

Values are means ± SD, BMI-body mass index.

**Table 2:** Changes (%) in body composition.

Body Parameter	6 Months	12 Months
Total body fat %	−2.2 ± 4.9	−1.7 ± 7.2
Total body fat (g)	−5.1 ± 7.8	−4.9 ± 11.0
Visceral adipose tissue	−8.0 ± 25.5	−3.8 ± 32.7
Total lean mass	−1.2 ± 3.3	−1.7 ± 3.8
Appendicular lean mass	−1.1 ± 8.0	−4.8 ± 6.0

The above table illustrates % changes in body composition. Data are presented as mean ± SD. Measurements were obtained at baseline and follow-up visits (6 and 12 months). *p*-values are not significant compared to baseline by Student’s *t*-test.

**Table 3:** Changes in methylation with lifestyle intervention ± aromatase inhibitors.

	LSI + AI	LSI + PBO	*p*	* *p*	** *p*
**Weight change (kg)**					
**6 months**	−5.8 ± 6.8	−3.8 ± 6.9	0.44		
**12 months**	−7.1 ± 7.8	−6.2 ± 6.3	0.72		
**5-mC%**					
**6 months**	−0.32 ± 0.39	−0.29 ± 0.55	0.86	0.71	
**12 months**	−0.17 ± 0.43	−0.08 ± 0.59	0.62	0.47	0.002
**%Global methylation**					
**6 months**	−0.05 ± 0.08	−0.01 ± 0.22	0.67	0.79	
**12 months**	−0.17 ± 0.16	−0.04 ± 0.21	0.12	0.12	<0.001

*p* adj—* *p* adjusted for the intake of metformin and ** *p* adjusted by repeated measures ANOVA across visits (6 and 12 months). Note: there was no difference due to treatment with an aromatase inhibitor or placebo (*p* = 0.12 for 5-mC% and *p* = 0.06 for global methylation), testosterone (*p* = 0.62 for 5-MC% and *p* = 0.21 for global methylation), or estradiol (*p* = 0.35 for 5-mC% and *p* = 0.92 for global methylation), and an independent difference due to weight (*p* = 0.02 for 5-mC% and *p* = 0.03 for global methylation). LSI + AI—lifestyle intervention + aromatase inhibitor; LSI + PBO—lifestyle intervention + placebo.

## Data Availability

The original contributions presented in this study are included in the article/Supplementary Materials. Further inquiries can be directed to the corresponding author(s).

## Supplementary Materials

The following supporting information can be downloaded at: https://www.mdpi.com/article/10.3390/metabo16030198/s1, Table S1: Key resources table; Table S2: Baseline characteristics of the subjects included vs. those not included.
