# Supplementary material for: Lifestyle Intervention Therapy Modulates Global DNA Methylation and Adipogenic Gene Expression in Severely Obese Hypogonadal Men
Source: Metabolites. 2026 Mar 16;16(3):198. doi: 10.3390/metabo16030198 (PMC13028031; doi:10.3390/metabo16030198)
Supplement: Supplementary file 1 [file metabolites-16-00198-s001.zip › metabolites-4169347-supplementary.pdf]

Supplementary Table S1: Key Resources table

REAGENT/RESOURCE	SOURCE	IDENTIFIER
**Chemicals, Peptides, Metabolites, and Recombinant Proteins**		
RNALater	ThermoFisher	Cat# AM7020
HPLC-grade water	ThermoFisher	Cat# W64
Halt Protease Inhibitor Cocktail (100X)	ThermoFisher	Cat# 78430
HEPES	Biotechne	Car# 7365-45-9
PBS	Corning	Cat# 21-040
**Critical Commercial Assays/Machines**		
RNeasy Plus Universal Mini Kit	QIAGEN	Cat# AM1928
TaqMan Universal PCR Master Mix	ThermoFisher	Cat# 4304437
SuperScript VILO cDNA kit	ThermoFischer	Cat# P5644825
Global DNA Methylation Assay Kit	Abcam	Cat# Ab233486
96 well plate for PCR	Applied Biosystems	Cat# N8010560
FastPrep 24–5G homogenizer	MP Biomedicals	Cat # 1606-1026
Nanodrop and Bioanalyzer 2100	Agilent Technologies	Cat# P 275
Real Time PCR system	Applied Biosystem-	Cat # F272520135
Accuwash	ThermoFisher	Cat# 888-7482AF
Digital incubator	Wards science	Cat # 03211213
Spectra max Ab plus	Molecular devices	Cat# ABP 00603
**Oligonucleotides**		
Taqman FAM Probe PPARG	ThermoFisher	Hs01115513_m1
Taqman FAM Probe CEBPa	ThermoFisher	Hs00269972_s1
Taqman FAM Probe FTO	ThermoFisher	Hs01057145_m1
Taqman FAM Probe DNMT1	ThermoFisher	Hs00945875_m1
Taqman FAM Probe DNMT3A	ThermoFisher	Hs01027162_m1
Taqman FAM Probe DNMT3B	ThermoFisher	Hs00171876_m1
Taqman VIC Probe 18s	ThermoFisher	Hs03928990_g1
**Software and Algorithms**		
Quantstudio design and analysis software 1.3.1	ThermoFisher	N/A
Endnote version 21	Clarivate Analytics	N/A
Biorender software	Toronto, Ontario, Canada	N/A
Graph pad prism software 9.0	Dotmatics, CA, USA	N/A

Supplemental Table S2. Baseline characteristics of the subjects included vs. those not included.

	Included (n=35)	Not included (n=86)	
Age	51.8±6.9	50.9±7.8	0.54
BMI	43.4±5.2	41.6±6.0	0.12
Weight	136.2±19.6	131.7±21.3	0.28
Racial background			0.75
White	18	47	
Blacks	17	39	
Testosterone	227.7±46.9	230.9±55.0	0.75
Estradiol	23.5±12.9	25.6±9.9	0.34
Values are means±SD			
